# Pressure‐volume analysis in athyroid patients off and on thyroxine supplementation: a pilot study

**DOI:** 10.14814/phy2.13883

**Published:** 2018-10-22

**Authors:** Marcelo B. Bastos, Elske T. Massolt, Boen L. R. Kam, Robin P. Peeters, Nicolas M. Van Mieghem, W. Edward Visser, Corstiaan A. den Uil

**Affiliations:** ^1^ Department of Cardiology Erasmus MC Rotterdam The Netherlands; ^2^ Division of Endocrinology Erasmus MC Rotterdam The Netherlands; ^3^ Rotterdam Thyroid Center Department of Internal Medicine Erasmus MC Rotterdam The Netherlands; ^4^ Department of Nuclear Medicine Erasmus MC Rotterdam The Netherlands; ^5^ Department of Intensive Care Erasmus MC Rotterdam The Netherlands

**Keywords:** Afterload, cardiac function, contractility, hemodynamics, hypothyroidism, preload, pressure‐volume analysis

## Abstract

Thyroid hormone importantly affects the cardiovascular system. However, evaluation of stroke volume (SV) and its determinants is confounded by variations in volume status that occur along different thyroid states. This study applied the pressure‐volume (PV) framework to obtain relatively load‐independent estimates of cardiac function in hypothyroidism as compared to euthyroidism. Ten athyroid patients were assessed echocardiographically after 4 weeks in deep hypothyroid state, and again after supplementation with oral Levothyroxine (LT4) for 3 months. Thyroid hormone levels were assessed and noninvasive pressure‐volume (PV) analysis based on dedicated repeated echocardiograms was performed. Changes were assessed using paired tests. Results are presented as medians and interquartile ranges. Hypothyroidism was associated with reduced stroke volume (SV: 67.6 ± 17 vs. 75.7 ± 20.6 mL,* P* = 0.024), preload (end‐diastolic volume, EDV: 122.6 ± 32.5 vs. 135.7 ± 33.6 mL,* P* = 0.004), and contractility (end‐systolic elastance, *E*_es_: 1.7 ± 0.33 vs. 2.58 ± 1.33 mmHg/mL,* P* = 0.01). Afterload was constant (effective arterial elastance, *E*_a_: 1.66 ± 0.32 vs. 1.79 ± 0.52 mmHg/mL,* P* = 0.43) and the total energy spent was lower (PVA∙HR: 86.7 ± 28 vs. 110.9 ± 32.1 J, *P* = 0.04). Hemodynamic manifestations of frank hypothyroidism in humans are characterized by reduced preload and contractility, and unchanged total afterload. LT4 therapy increased work efficiency and heart rate, but not the net energy expenditure. Noninvasive PV analysis may be useful to follow‐up different thyroid states.

## Introduction

Thyroid hormone (TH) has important cardiovascular effects. Thyroid dysfunction is a well‐recognized risk factor for many cardiovascular diseases. Hyperthyroidism is associated with increased risk of atrial fibrillation (AF) as well as sudden cardiac death (Collet et al. 2012; Chaker et al. 2016). Thyroid storm is an endocrine emergency because of the risk of cardiac failure. Hypothyroidism is associated with an increased risk of coronary heart disease events and mortality (Cappola and Ladenson 2003; Rodondi et al. 2010).

The pathophysiological mechanisms behind these clinical well‐established associations are not fully elucidated. The chronotropic effects of TH underlie the presence of sinus tachycardia at rest and occasionally AF in hyperthyroidism. Left ventricle (LV) hypertrophy and left atrium (LA) enlargement are part of the initial manifestations of hyperthyroidism, later progressing to rate‐related systolic dysfunction if left untreated (Iwasaki et al. 1989). Furthermore, hyperthyroid patients, whose cardiac output (CO) tends to be on average 50–300% above normal, often have systolic hypertension (Klein and Ojamaa 2001). At the metabolic level, a less efficient use of the products of oxidative metabolism leads to energetic deficit and oxidative stress while facing the higher demands posed by this hyperdynamic state (Maity et al. 2013). In contrast, the abovementioned effects are mostly the opposite in hypothyroidism, including reduced CO and elevation of the diastolic blood pressure relative to systolic. Ventricular ejection time (ET), stroke volume (SV), end‐diastolic volume (EDV), and circulating blood volume tend to decrease (Wieshammer et al. 1988; Monzani et al. 2001).

The assessment of the physiological effects of TH on the circulation faces several difficulties. Evaluation of the contractile function in thyroid dysfunction is confounded by variations in volume status occurring along the serial assessments under different thyroid states. Diverse studies have attempted to assess the determinants of the hypodynamic state in hypothyroidism, with only partial reproducibility of results (Forfar et al. 1985; Wieshammer et al. 1988; Monzani et al. 2001; Hoftijzer et al. 2009). One study documented increases in the maximal systolic elastance (*E*_max_) in subclinical hypothyroidism following subtotal thyroidectomy, but used vasodilators on the process and hence could not draw conclusions on the afterload (Forfar et al. 1985). One second study on hypothyroidism reported no increase in *E*_max_, but 44% of the nine patients of this study were not in hypothyroidism during the measurements (Wieshammer et al. 1988). Notwithstanding, in vitro and animal studies consistently reported TH to have biomolecular mechanisms that influence contractility (Taylor et al. 1969; Strauer and Schulze 1976).

The pressure‐volume (PV) framework is a time‐varying elastance model inspired on Otto Frank`s first descriptions of the cardiac PV relations in conjunction with Starling`s Law of the Heart, Sarnoff`s ventricular function curve, and Sonnenblick`s force–velocity curve. Inferences on contractility were firstly validated in canine excised hearts by H. Suga in 1969 and later extended to ventricular energetics. Based on these developments, the *E*_max_ and the pressure‐volume area (PVA) became widely popular as load‐independent indices of the cardiac function. When used invasively, the PV framework is today the gold standard for evaluating the cardiac function as it minimizes the effects of variations in loading conditions over the estimations. Further ahead, single‐beat algorithms developed to estimate systolic and diastolic relations extended the framework reach to the noninvasive spectra (Chen et al. 2001; Klotz et al. 2006).

This is an interesting perspective in the context of the cardiac effects of THs. Most studies on this regard lacked load‐independent estimates of cardiac function or applied it partially. The present study provides the first noninvasive load‐independent evaluation of cardiac function using state‐of‐the‐art PV analysis in patients exposed to a standardized period of deep hypothyroidism followed by thyroxine (LT4) supplementation.

## Methods

### Subjects

Patients of 18–70 years old with differentiated thyroid cancer (DTC) were recruited from the outpatient clinic of the Erasmus Medical Center Rotterdam, between June 2015 and March 2016. Initial therapy consisted of total thyroidectomy. Patients were eligible for inclusion if they were scheduled for ablation with radioactive iodine (RAI) and did not have heart failure or drugs interfering with TH metabolism. The first measurement was scheduled after 4 weeks off LT4 (i.e., LT4 withdrawal before RAI ablation) and the second measurement after 3 months on LT4 replacement therapy once TSH (thyroid‐stimulating hormone) suppression was reached. The Medical Ethics Committee of the Erasmus Medical Center approved the study protocol (MEC‐2014‐489) and written informed consent was obtained from all study participants.

### Laboratory measurements

Serum‐free T4 (FT4), total T4, and total T3 concentrations were measured by chemoluminescence assays (Vitros ECI Immunodiagnostic System; Ortho‐Clinical Diagnostics, Rochester, MI). Serum TSH was measured by immunometric assay (Immulite 2000 XPi, Siemens, The Hague, the Netherlands).

### Cardiac function

Transthoracic echocardiography using an iE33 Ultrasound System (Philips Healthcare, Amsterdam, the Netherlands) was performed by experienced technicians who were not aware of the thyroid state of the subjects. Preload was assessed before and after LT4 replacement through measurements of left atrial volume (LAV) and left ventricular end‐diastolic volume (EDV), obtained with biplane Simpson's method (TomTec Cardiac Performance Analysis, Munich, Germany). The end‐diastolic pressure (EDP) was estimated using the Nagueh formula. Total afterload was evaluated before and after LT4 replacement as the effective arterial elastance (*E*_a_) using available algorithms for noninvasive PV analysis (Sunagawa et al. 1983; Chen et al. 2001). Systemic vascular resistance (SVR) was estimated as 80(MAP ‐ RAP)/CO, where RAP represents the Right atrial pressure and CO the cardiac output. Contractility was represented by end‐systolic pressure‐volume relationship (ESPVR) and chamber stiffness by the *β* coefficient, both assessed using noninvasive PV analysis. To obtain the end‐systolic elastance (*E*_es_), the elastance at mid‐isovolumic contraction (Ed) is derived using systolic time intervals and the diastolic blood pressure. Subsequently, the estimated value of Ed normalized to the end‐systolic elastance was calculated and used to derive the *E*_es_. *β* was calculated using a specific algorithm obtained from pressure and volume‐normalized EDPVRs obtained from 80 ex vivo human hearts with different pathologies (Chen et al. 2001; Klotz et al. 2006). Additional assessment of the cardiac function included ejection fraction (EF), stroke volume (SV), and cardiac power output (CPO), given by CPO=(MAP∙CO)/451. Early and late transmitral inflow velocities (E and A waves) and deceleration time (DET) were measured from the mitral inflow pattern derived from the pulsed‐wave Doppler, from which the E/A ratio was calculated. Systolic time intervals (isovolumic contraction time, ICT; and ejection time, ET) were obtained from tissue Doppler of the medial mitral annulus and from pulsed Doppler of the LV outflow tract, with end‐systole set to the peak of the R‐wave. Ventricular‐arterial coupling (VAC) was assessed by means of the *E*_a_/*E*_es_ ratio. Pressure‐volume area (PVA) and stroke work (SW), reflecting respectively the total energy available for each cardiac cycle and the work done by the ventricle to eject the stroke volume, were obtained. Their ratio, reflecting the rate of PVA to SW conversion (work efficiency fraction, WEF), was derived. The ratio of cardiac output (CO) to PVA∙HR (to account for 1 min of PVA) was compared prior and after treatment to evaluate the effect of the chronotropic changes.

### Statistical analysis

Data are expressed as median (IQR) or mean (SD) as appropriate. Parametric data were compared with Student t‐tests and nonparametric data with Wilcoxon signed rank tests between patients off and on LT4 treatment. Spearman *ρ* was used to assess correlations between changes in *E*_es_ and changes in TH levels. The R v3.3.3 package was applied with type I error set to 5%. Multiplicity corrections were not applied because this study was exploratory and most of its variables are correlated (Bender and Lange 2001; Schulz and Grimes 2005).

## Results

**Figure 1:**
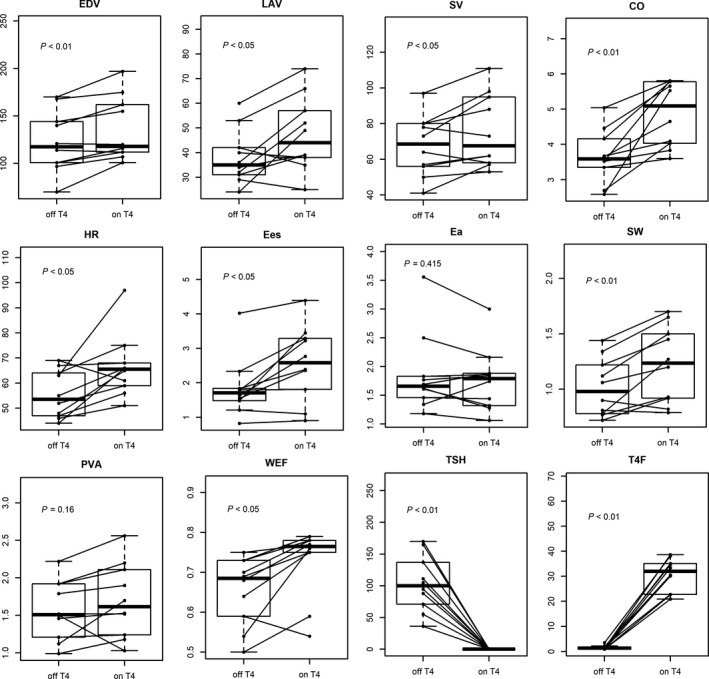
Comparison of hemodynamic variables (box plots) off and on T4. HR, heart rate; CO, cardiac output; EDV, end‐diastolic volume; LAV, left atrial volume; PVA, pressure‐volume area; *E*_es_, end‐systolic elastance; SW, stroke work; WEF, work efficiency fraction; *E*_a_, arterial elastance; SV, stroke volume; TSH, thyroid‐stimulating hormone; T4F, serum‐free T4.

Baseline characteristics are described in Table 1. Measurements are described in Table 2 and Figure 1. As anticipated, in deep hypothyroidism, T3 and FT4 were significantly depressed while TSH was high. Heart rate (HR) was significantly reduced pretreatment, as well as SV (mean: 68 ± 17 vs. 76 ± 21 mL, *P* = 0.024).

**Table 1:** Characteristics of study participants (*n* = 10). Data are expressed as median (IQR) or as percentages

Baseline characteristics	
Female sex *N* (%)	6 (60)
Age (years)	43 (30–56)
Time between tests (weeks)	12 (11–16)
Dose LT4 (*μ*g)	188 (150–225)
Dose LT4 (*μ*g/kg)	2.1 (1.8–2.6)
BMI (kg/m²)	28.0 (23.0–31.8)

**Table 2:** Results (means ± SD or medians ± IQR as adequate)

Variable	Hypothyroidism	Euthyroidism	*P*‐value	Normal values
Diastolic function				
*E*‐wave (cm/sec)	0.58 ± 0.21	0.71 ± 0.27	0.03	60–91
*E*/*A*	1.38 ± 1.08	1.31 ± 0.80	0.22	0.73–1.53
*E*/*E*′	8.18 ± 2.45	8.82 ± 1.45	0.48	<12
DET (msec)	195 ± 56	196 ± 72	0.54	138–219
DBP (mmHg)	83.20 ± 9.70	79.10 ± 7.81	0.26	60–89
EDV (mL)	122.6 ± 32.49	135.7 ± 33.59	<0.01	29–74
EDP (mmHg)	12.25 ± 1.94	12.94 ± 1.47	0.47	4–12
Systolic function				
ICT (msec)	58 (49 to 68)	23 (19 to 28)	<0.01	23–49
ET (msec)	262 ± 29	282 ± 23	<0.001	257–314
ICT/ET	0.20 (0.18 to 0.27)	0.08 (0.06 to 0.10)	<0.01	0.07–0.17
SV (mL)	68 ± 19	76 ± 21	0.024	79–131
EF (%)	57 ± 5	56 ± 5	0.95	52–74
SBP (mmHg)	129 (117 to 137)	133 (127 to 141)	0.036	90–140
Global assessment				
NT–pro–BNP (pg/mL)	1.0 ± 0.0	6.0 ± 4.0	0.01	<125
LAV (mL)	38.1 ± 11.1	47.4 ± 15.1	0.027	16–34
RAP (mmHg)	4 (3 to 5)	5 (5 to 5)	0.072	1 to 8
HR (bpm)	55.5 ± 9.47	66.4 ± 12.67	0.015	60–100
CPO (W)	1.5 ± 0.34	2.09 ± 0.57	<0.01	>0.6
SVR (dyn·sec/cm^5^)	1972 ± 587.01	1626 ± 446.48	<0.01	900–1400
Pressure‐volume analysis				
SW (J)	1.02 ± 0.26	1.22 ± 0.34	<0.01	No data
VAC	0.91 (0.79 to 1.32)	0.62 (0.57 to 0.68)	0.03	0.62–1.30
PVA (J)	1.56 ± 0.39	1.70 ± 0.49	0.15	No consensus
SV/PVA (mL/J)	42.88 ± 7.98	44.34 ± 6.54	0.5	No consensus
PVA∙HR (J/min)	86.86 ± 28.02	110.89 ± 32.05	0.04	No consensus
*E*_es_ (mmHg/mL)	1.70 (1.50 to 1.83)	2.58 (1.95 to 3.27)	0.01	2.0–4.0
*V*_o_ (mL)	−12.9 (−30.2 to −2.9)	10.0 (5.7 to 18.8)	0.01	No consensus
*E*_a_ (mmHg/mL)	1.66 (1.50 to 1.82)	1.79 (1.35 to 1.88)	0.43	1.4–3.0
WEF	0.68 (0.60 to 0.72)	0.76 (0.75 to 0.78)	0.01	No consensus
β	3.02 ± 0.05	3.04 ± 0.04	0.47	No consensus
Thyroid function				
TSH (mU/L)	99.80 (75.25 to 130.50)	0.07 (0.02 to 0.20)	<0.01	0.4–4.3
Free T4 (pmol/L)	1.3 (0.9 to 1.8)	32.0 (24.6 to 35.0)	<0.01	11–25
T3 (nmol/L)	0.63 ± 0.33	2.01 ± 0.39	<0.01	1.4–2.5
Total T4 (nmol/L)	13 ± 7	163 ± 44	<0.01	58–128

E‐top, early transmitral inflow velocity; A, late transmitral flow velocity; E′, early diastolic mitral annular velocity; DET, decelerating time; SBP, systolic blood pressure; ICT, isovolumic contraction time; ET, ejection time; SV, stroke volume; EF, ejection fraction; DBP, diastolic blood pressure; NT‐pro‐BNP, brain natriuretic peptide; EDV, end‐diastolic volume; LAV, left atrial volume; RAP, right atrial pressure; HR, heart rate; CPO, cardiac power output; SVR, systemic vascular resistance; SW, stroke work; VAC, ventricular–arterial coupling ratio; PVA, pressure‐volume area; *E*_es_, end‐systolic elastance; *V*_o_, unstressed volume; *E*_a_, arterial elastance; WEF, work efficiency fraction; β, chamber stiffness constant; TSH, thyroid‐stimulating hormone.

During hypothyroidism, markers of preload (i.e., E‐wave velocity and filling volumes EDV and LAV) were significantly reduced. Also, NT‐pro‐BNP levels were significantly reduced. The E‐wave was significantly lower but the *E*/*A* and *E*/e′ ratios were unchanged, suggesting that the reduced preload in hypothyroidism decreased the magnitude of the mitral inflow.

**Figure 2:**
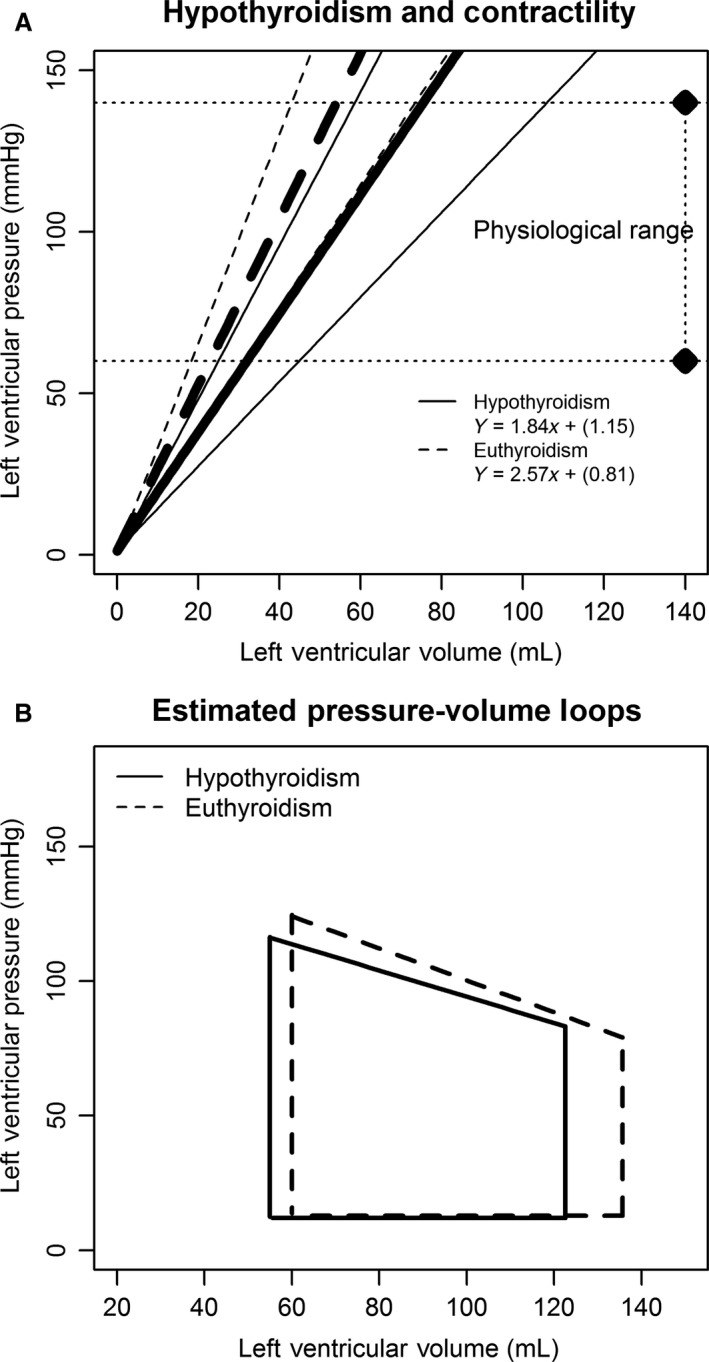
(A) Change in the systolic function. In hypothyroidism, the ESPVR assumes a smaller slope indicating that the ventricles are producing less pressure under the same ranges of volume. ESPVR regression lines are derived from mean coefficients of *E*_es_ and *V*_o_ and 95% confidence intervals. ESPVR, end‐systolic pressure‐volume relationship. (B) Estimated pressure‐volume loops summarizing hemodynamic changes between the two states.

**Figure 3:**
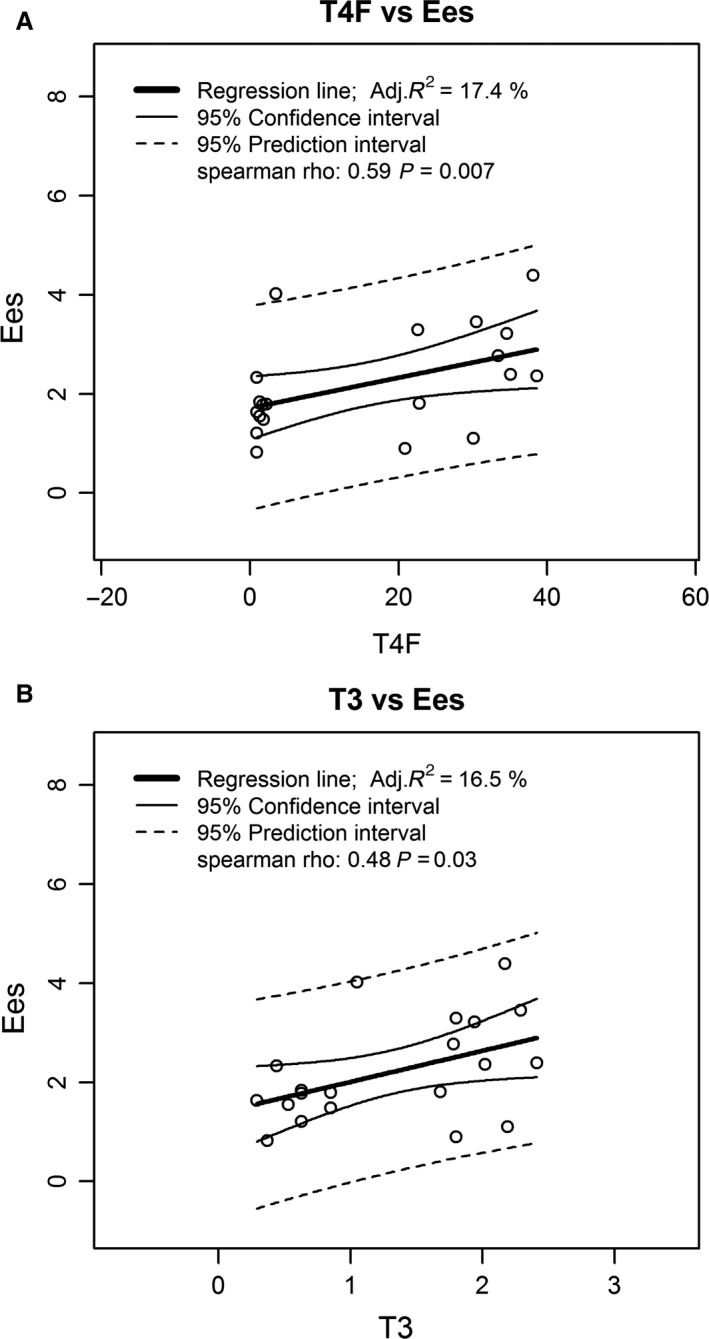
Analysis of correlations between *E*_es_ and thyroid hormones. *E*_es_, end‐systolic elastance.

Contractility was decreased in hypothyroidism, as the *E*_es_ was significantly lower and the end‐systolic pressure‐volume relationship (ESPVR) was shifted to the right and downwards (Fig. 2). *E*_es_ showed a positive monotonic correlation with FT4 levels (*ρ* = 0.59, *P* = 0.007) and with T3 levels (*ρ* = 0.48, *P* = 0.03), as exhibited in Figure 3. Ejection fraction (EF) remained equivalent, while CPO was significantly decreased pretreatment.

SVR was increased in hypothyroidism, but SBP was significantly lower. As a consequence, the total afterload (*E*_a_) was not different from euthyroidism. Nevertheless, as contractility was lower, the balance of forces favoring and opposing blood ejection into the ascending aorta (VAC) was significantly worse (i.e., <1.0) in the hypothyroid state.

The PVA remained equal at beat level, but when adjusted for HR, it was significantly lower in hypothyroidism. Work efficiency was also decreased as SW was reduced and occupied a lower proportion of the total energy available (WEF, SW/PVA ratio). This indicates less mobilization of the myocardial energy. Conversely, the SV/PVA ratio was unchanged suggesting that after correction, the fraction of SV displaced by 1J of PVA remained the same.

## Discussion

This is the first study measuring full‐scale changes in the cardiac function of patients with deep hypothyroidism versus high‐normal thyroid states, applying noninvasive, relatively load independent, PV analysis (Table 3). Previous attempts to apply such techniques in this setting date back to 1985 and 1988. Since then, PV theories have evolved considerably. This study describes promising data and introduces a new follow‐up tool for thyroid disease that calls for further confirmatory studies. LT4 supplementation improved the hemodynamic profile and led to better ventricular–arterial coupling by increasing preload and contractility while keeping total afterload equal. Despite improved efficiency at beat level, chronotropic increments resulted in higher energetic costs in euthyroidism.

**Table 3:** Previous studies on cardiac function in hypothyroidism applied different techniques. Two studies used SBP/SV when PV theory was on its first steps (see introduction) and reported divergent findings

Year	Author	References	Population	Condition	*n*	Contractility Index	Preload	Contractility	Afterload
1969	Taylor	(Taylor et al. 1969)	Dog	DH	15	TVR	↓	↓	NR
1976	Strauer	(Strauer and Schulze 1976)	Cat	DH	82	TVR and +dP/dt_max_	NR	↓	NR
1977	Crowley	(Crowley et al. 1977)	Human	DH	15	STI	NR	↓	NR
1985	Sharp	(Sharp et al. 1985)	Rabbit	DH	33	Time to Peak Tension	NR	↓	NR
1985	Forfar	(Forfar et al. 1985)	Human	SH	10	SBP/ESV ratio	NR	↓	NR
1988	Wieshammer	(Wieshammer et al. 1988)	Human	DH	9	Noninvasive SBP/ESV ratio	↑	↔	↓
2001	Monzani	(Monzani et al. 2001)	Human	SH	20	Ultrasonic videodensitometry	↔	↓	↔
2009	Hoftijzer	(Hoftijzer et al. 2009)	Human	DH	14	FS and EF	↑	↔	NR

DH, deep hypothyroidism; EF, ejection fraction; ESV, end‐systolic volume; NR, not reported; SBP, systolic blood pressure; SH, subclinical hypothyroidism; STI, systolic time intervals; TVR, tension–velocity relation.

### Global determinants of stroke volume

The three main determinants of SV consist of preload, afterload, and contractility. During hypothyroidism, the decreased LV and LA volumes in conjunction with lower pro‐BNP concentrations and lower passive mitral inflow velocities indicate a reduction in preload, which is in line with previous results (Buccino et al. 1967; Biondi et al. 2002). Afterload is a complex concept that is difficult to evaluate in vivo. Several studies used different measurement techniques, looking at the various components of the total afterload. These approaches cannot be considered separately from other parameters (Chirinos and Segers 2010). To exemplify, the diastolic aortic pressure opposes the start of ejection, directly affecting systolic time intervals and hence contractility (Hassan and Turner 1983). The distal bed, mostly composed by small caliber vessels measuring less than 500 *μ*m diameter, can be better evaluated by the SVR, which represents the steady component of the arterial load (Nichols et al. 1977; Mitchell et al. 2001; Chirinos et al. 2012). The aortic impedance reflects the pulsatile component of the arterial load in the ascending aorta. The total arterial compliance (TAC) responds for the pulsatile component in the descending aorta, in large arteries, and to a lesser degree in smaller vessels (Stergiopulos et al. 1999). The total afterload denotes the summation of the steady and pulsatile components.

### Findings from PV analysis

#### Effective arterial elastance

*E*_a_, the ratio between the end‐systolic pressure (ESP) and SV, has been proposed to represent the net arterial load against which blood is ejected, thus reflecting the resultant of the abovementioned variables. It has the advantage of being readily comparable with the *E*_es_, a measure of contractility (Sunagawa et al. 1983; Klein and Ojamaa 2001). It can also be described as *E*_a_ = SVR/(60/HR) + 0.42/C–0.04, where C represents TAC (Chemla et al. 2003), showing significant agreement with the impedance spectra. *E*_a_ integrates the two components of the arterial load (Kelly et al. 1992). In the current study, LT4 supplementation reduced SVR without changing *E*_a_ and DBP. This may reflect fluid retention and chronotropic increments, turning the arterial system into a high impedance hypocompliant compartment, and counterposing a decreased steady component. The significantly increased SBP, which is in consonance with this pattern, contributes in keeping *E*_a_ constant.

#### Systolic time intervals

Hypothyroidism decreased contractility due to both lower velocity of fiber shortening and lower systolic pressure buildup. This is demonstrated by our findings that ICT and *E*_es_ were significantly smaller and the ICT/ET ratio was significantly higher in euthyroidism. These findings are in line with previous studies comparing the pre‐ejection period (PEP) with ET under different thyroid states (Crowley et al. 1977). Of note, since the intraventricular conduction times were statistically the same, this analysis only applied the mechanical constituent of PEP (i.e., ICT).

#### Ventricular–arterial coupling and energetics

VAC, which represents the balance between contractility and afterload, was significantly worse under hypothyroid conditions. While maximal efficiency has reportedly been linked to a ratio equal to or slightly below 1.0 (De Tombe et al. 1993), our results demonstrate that LT4 treatment facilitated the production of antegrade flow.

SW quantifies the conversion of chemomechanical energy stored in the myocardium into hydraulic energy during each heart cycle, while CPO predicts worse prognosis in heart failure when markedly reduced. Both measurements significantly decreased in hypothyroidism. SW reduction was due to decreases in both systolic pressure and SV.

At beat level, although the pressure‐volume area (PVA) was constant, WEF was higher in euthyroidism. This indicates better mobilization of the metabolic and viscoelastic energetic reserves at myocardial level at the same total energetic cost. Contractility changes after LT4 replacement are demonstrated by up‐shifting of the ESPVR and by a significant increment on the slope *E*_es_. Although increased contractility and SV would be expected to elevate MVO_2_ at beat level, that did not happen possibly due to the observed TH‐mediated preload changes, which may passively assist LV function, as previously postulated (Biondi et al. 2002).

Higher values of WEF and increased SV occurred at similar SV/PVA ratio indicating an improvement in both work efficiency and flow production. Nevertheless, when accounting for the increased HR, the net amount of energy available during 1 min, estimated in joules of PVA (PVA∙HR), was higher posttreatment. Hence, as a result of the chronotropic response to TH, the efficiency gain verified at beat level was not translated into a less energetically costly cardiovascular state.

### Strengths and limitations

Limitations of this study consist on small sample size, shortness of exposure periods, and absence of tonometric measurements that would warrant a more precise quantification of the pulsatile component of the afterload. Also strain analysis was not possible. The study is exploratory and its findings require further confirmation prior to implementation in clinical practice. Strengths consist in a fully integrated cardiac and global hemodynamic assessment with well‐established and state‐of‐the‐art tools, minimizing the bias derived from the variation in fluid status. In addition, the use of paired analysis to reduce confounding should be mentioned, as well as the entirely noninvasive way of data collection using dedicated echocardiography.

## Conclusion

In conclusion, in patients with deep hypothyroidism, LT4 therapy increases contractility and preload, while the total afterload remained constant. The new state was more energetically efficient at beat level and theoretically explains the increase in systolic pressures. Positive chronotropism, however, elevates CO and the net energetic cost in 1 min. The results indicate for the first time that integrated pressure‐volume analysis can be noninvasively applied in the follow‐up of patients using levothyroxine. A large‐scale study screening patients with noninvasive PV analysis on different phases of thyroid disease would be desirable to characterize this technique as a predictive tool for systolic dysfunction and other cardiovascular outcomes. Also, a clearer demonstration of how the different components of afterload would respond to TH would be adequate. The obtained information should be useful for tailoring therapeutics.

## Conflict of Interest

The abovementioned authors have no conflicts to disclose.
